# Supplementary material for: Spring flowering habit in field pennycress (Thlaspi arvense) has arisen multiple independent times
Source: Plant Direct. 2018 Nov 15;2(11):e00097. doi: 10.1002/pld3.97 (PMC6508777; doi:10.1002/pld3.97)
Supplement: Supplementary file 1 [file PLD3-2-e00097-s002.docx]

**Supporting Information Brief Legends**

**Fig. S1 -** PCR strategy for identifying *flc-B* allele

**Fig. S2 -** PCR strategy for identifying *flc-C* allele

**Fig. S3 -** Peptide alignment of Brassicaceae FRIGIDA predicted peptides and pennycress FRI gene model

**Fig. S4 -** Peptide alignment of Brassicaceae FLOWERING LOCUS C predicted peptides

**Fig. S5 -** Genomic DNA alignment of pennycress FLOWER LOCUS C wild type and *flc-a* allele

**Fig. S6 -** Identification and characterization of pennycress flc-α

**Table S1 -** Summary of whole genome sequencing reads of pennycress accessions used in this study

**Table S2 -** Primer sequences used in PCR experiments used in this study

**Table S3 -** Summary of pennycress accessions and the corresponding alleles of FLC as identified in this study
