# Supplementary figures and images for: Spring flowering habit in field pennycress (Thlaspi arvense) has arisen multiple independent times
Source: Plant Direct. 2018 Nov 15;2(11):e00097. doi: 10.1002/pld3.97 (PMC6508777; doi:10.1002/pld3.97)

**A**

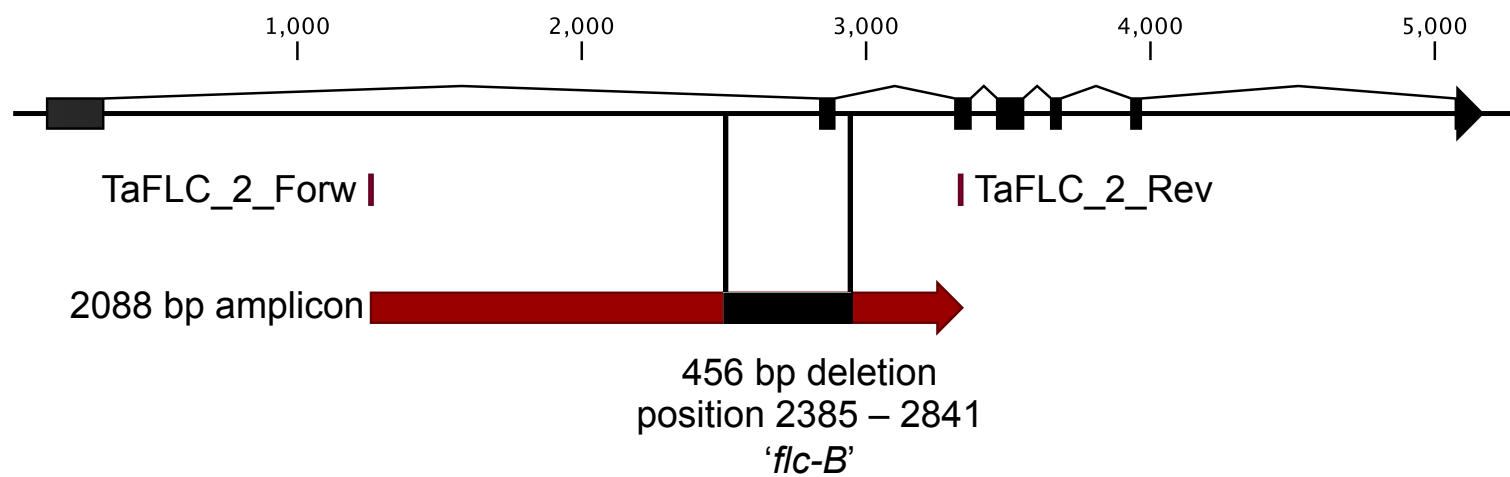

Supplement: Supplementary file 2 [file PLD3-2-e00097-s001.pdf]

**A**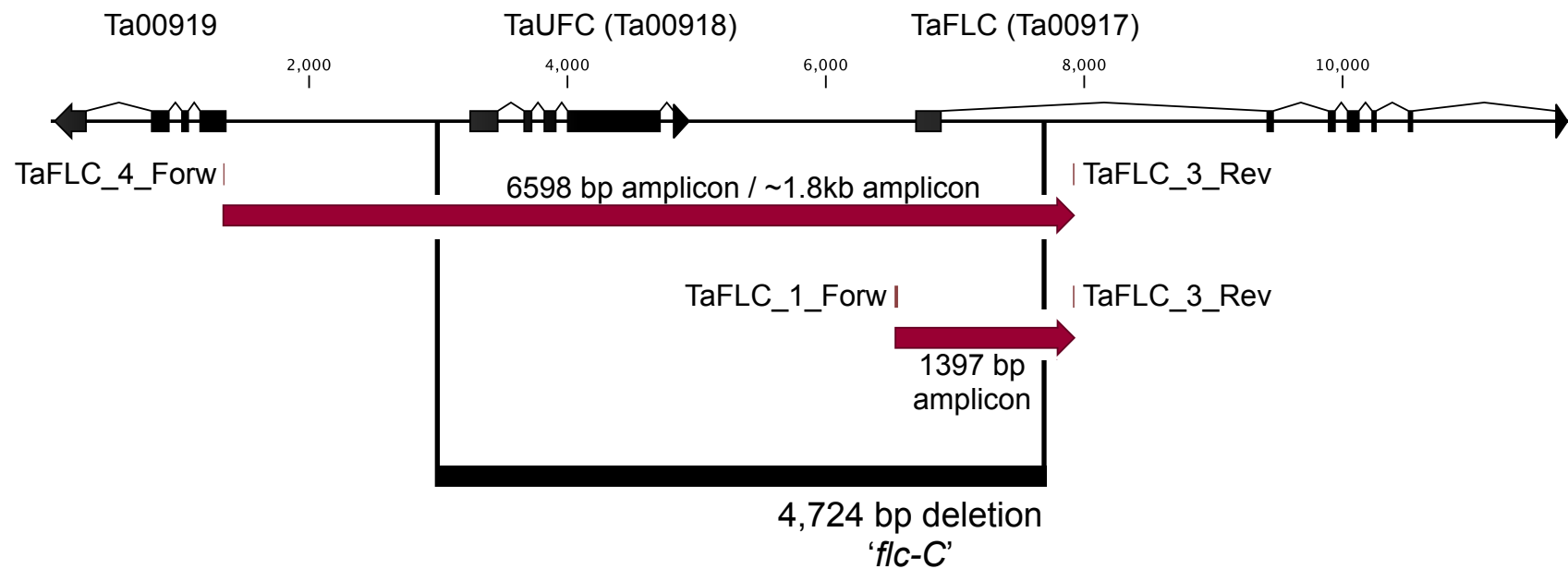

Supplement: Supplementary file 3 [file PLD3-2-e00097-s003.pdf]
