# Supplementary material for: Spring flowering habit in field pennycress (Thlaspi arvense) has arisen multiple independent times
Source: Plant Direct. 2018 Nov 15;2(11):e00097. doi: 10.1002/pld3.97 (PMC6508777; doi:10.1002/pld3.97)
Supplement: Supplementary file 4 [file PLD3-2-e00097-s004.pdf]

A

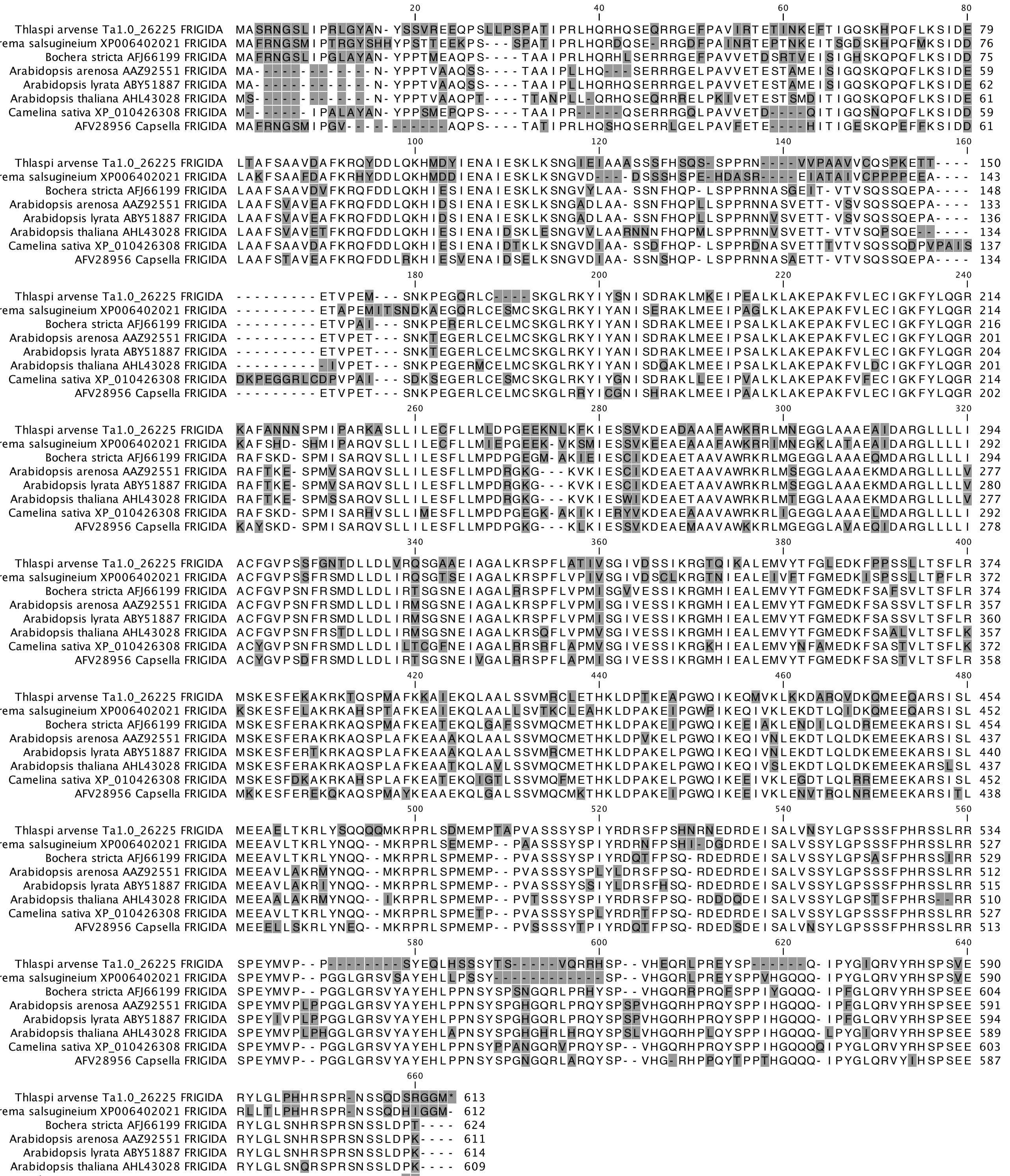
Thlaspi arvense Ta1.0_26225 FRIGIDA
Eutrema salsugineum XP006402021 FRIGIDA
Bochera stricta AFJ66199 FRIGIDA
Arabidopsis arenosa AAZ92551 FRIGIDA
Arabidopsis lyrata ABY51887 FRIGIDA
Arabidopsis thaliana AHL43028 FRIGIDA
Camelina sativa XP_010426308 FRIGIDA
AFV28956 Capsella FRIGIDA

B

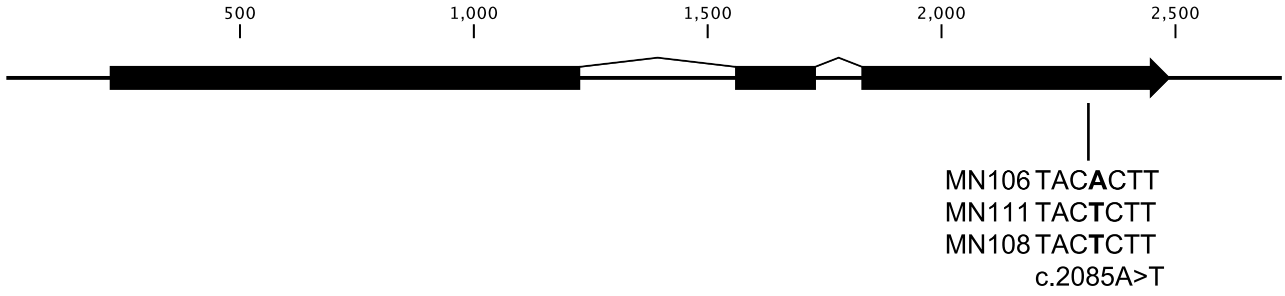
500
1,000
1,500
2,000
2,500
MN106 TACACTT
MN111 TACTCTT
MN108 TACTCTT
c.2085A>T
