# Supplementary material for: Spring flowering habit in field pennycress (Thlaspi arvense) has arisen multiple independent times
Source: Plant Direct. 2018 Nov 15;2(11):e00097. doi: 10.1002/pld3.97 (PMC6508777; doi:10.1002/pld3.97)
Supplement: Supplementary file 5 [file PLD3-2-e00097-s005.pdf]

A

```
                                                        20                  40                  60
Thlaspi arvense MN106 FLC          MGRKKLEIKRIENKSSRQVTFSKRRNGLIEKARQLSVLCDASVALLVVSASGKLYSFSSGDKYDFSSSGLVKILD 75
Thlaspi arvense MN111 FLC          MGRKKLEIKRIENKSSRQVTFSKRRNGLIEKARQLSVLCDASVALLVVSASGKLYSFSSGDKYDFSSSGLVKILD 75
Thlaspi arvense MN108 FLC          MGEKKTRNQAN---------------------------------------------------------------- 11
Arabidopsis thaliana FLC           MGRKKLEIKRIENKSSRQVTFSKRRNGLIEKARQLSVLCDASVALLVVSASGKLYSFSSGDN-------LVKILD 68
Capsella rubella FLC AFV28913.1    MGRKKLEIKRIENKSSRQVTFSKRRNGLIEKARQLSVLCDASVALLVVSASGKLYSFSSGDN-------LVKILD 68
Arabidopsis lyrata FLCAFV33319     MGRKKLEIKRIENKSSRQVTFSKRRNGLIEKARQLSVLCDASVALLVVSASGKLYSFSSGDN-------LVKILD 68
Camelina sativa FLC XP_010453038   MGRKKLEIKRIENKSSRQVTFSKRRNGLIEKARQLSVLCDASVALLVVSASGKLYSFSSGDN-------LVKILD 68
Eutrema halophilum FLC AY957537    MGRKKLEIKRIENKSSRQVTFSKRRNGLIEKARQLSVLCDASVALLVVSASGKLYSFSSGDN-------LVKILD 68
Brassica rapa FLC ABI29999         MGRKKLEIKRIENKSSRQVTFSKRRNGLIEKARQLSVLCDASVALLVVSASGKLYSFSSGDN-------LVKILD 68
Brassica napus FLC AAK70215        MGRKKLEIKRIENKSSRQVTFSKRRNGLIEKARQLSVLCDASVALLVVSASGKLYSFSSGDN-------LVKILD 68
Arabis alpina PEP1 ACQ44228        MGRKKLEIKRIENKSSRQVTFSKRRNGLIEKARQLSVLCDASVALLVVSSSGKLYSFSSGDN-------LVKILD 68

                                        80                 100                 120                 140
Thlaspi arvense MN106 FLC          RYGKQHADDLKALDLQSKALNYGSHHELLELVESKLVESDVDNVSVDSLVQLEDHLETALSITRARKTELMLKLV 150
Thlaspi arvense MN111 FLC          RYGKQHADDLKALDLQSKALNYGSHHELLELVERKLVESDVDNVSVDSLVQLEDHLETALSITRARKTELMLKLV 150
Thlaspi arvense MN108 FLC          --------------------------------------------------------------------------- 11
Arabidopsis thaliana FLC           RYGKQHADDLKALDHQSKALNYGSHYELLELVDSKLVGSNVKNVSIDALVQLEEHLETALSVTRAKKTELMLKLV 143
Capsella rubella FLC AFV28913.1    RYGKQHADGLKALDLQSKSLNYGSHHELLDLVESKLVESNVSNVSVDSLVQLEEHLETALSVTRAKKTELMLKLV 143
Arabidopsis lyrata FLCAFV33319     RYGKQHADDLKALDMQSKALNYGSHHELLELVESKLVGSNVNNVSAETLLQLEKHLETALSVTRAKKTELMLKLV 143
Camelina sativa FLC XP_010453038   RYGKQHADDLKALDLQSKALNYGSHHELLELVESNLVESNVNNVSVDDLVQLEEHLETALSVTRAKKTELILKLV 143
Eutrema halophilum FLC AY957537    RYGKQHADDLKALDLQSKALSYGSHHELLELVESQLVDSSVDNASVDSLAQLEDHLETALSVTRARKTELMLKLV 143
Brassica rapa FLC ABI29999         RYGKQHDDDLKALDRQSKALDCGSHHELLELVEGKLEESNVDNVSVGSLVQLEEHLENALSVTRARKTELMLKLV 143
Brassica napus FLC AAK70215        RYGKQHDDDLKALDRQSKALDCGSHHELLELVESKLEESNVDNVSVGSLVQLEEHLENALSVTRARKTELMLKLV 143
Arabis alpina PEP1 ACQ44228        RYGKRHADDLKALDLQSKALNYGSHHELLEIVESKLVAPNVNNVSFDTLVQLEKHLETALAVVRAKKTELMLKLL 143

                                            160                 180                 200
Thlaspi arvense MN106 FLC          DSLKEKEKLLKEENQVLASQMEKN-HMEADAD-NMEMSPGQISDMNLPVTLPLLN*--------- 204
Thlaspi arvense MN111 FLC          DSLKEKEKLLKEENQVLASQMEKN-HMEADAD-NMEMSPGQISDMNLPVTLPLLN---------- 203
Thlaspi arvense MN108 FLC          ----------------------------------------------------------------- 11
Arabidopsis thaliana FLC           ENLKEKEKMLKEENQVLASQMENNHHVGAEA--EMEMSPAGQISDNLPVTLPLLN---------- 196
Capsella rubella FLC AFV28913.1    ENLKEKEKLLKEENQVLASQMGKNHVVVGAETEMMEMSPAGQISDNLPVTLPLLN---------- 198
Arabidopsis lyrata FLCAFV33319     ENLKEKEKLLKEENQVLASQMEKNHHVGAEA--EMEISPAGQISDNLPVTLPLLN---------- 196
Camelina sativa FLC XP_010453038   ENLKEKEKLLKEENQVLARQMETNHVVGAEADMEMEMSPAGQISDNLPVTLPLLN---------- 198
Eutrema halophilum FLC AY957537    DSLKEKEKLLKRENQVLASQMEKNQHVGAEAD-NMEMSPGQISDSNLPVTLPLLN---------- 197
Brassica rapa FLC ABI29999         ENLKEKEKLLEEENHVLASQMEKSNLVRAEAD-NMDVSPGQISDINLPVTLPLLKGGLVGVEFL 206
Brassica napus FLC AAK70215        ENLKEKEKLLEEENHVLASQMEKSNLVRAEAD-NMDVSPGQISDINLPVTLPLLN---------- 197
Arabis alpina PEP1 ACQ44228        ESLKEKEKLLKEENQVLASQMEKKTLVGAEADDNMEISPGEISDINLPVTLPLLN---------- 198
```
