# Supplementary material for: Spring flowering habit in field pennycress (Thlaspi arvense) has arisen multiple independent times
Source: Plant Direct. 2018 Nov 15;2(11):e00097. doi: 10.1002/pld3.97 (PMC6508777; doi:10.1002/pld3.97)
Supplement: Supplementary file 6 [file PLD3-2-e00097-s006.pdf]

A

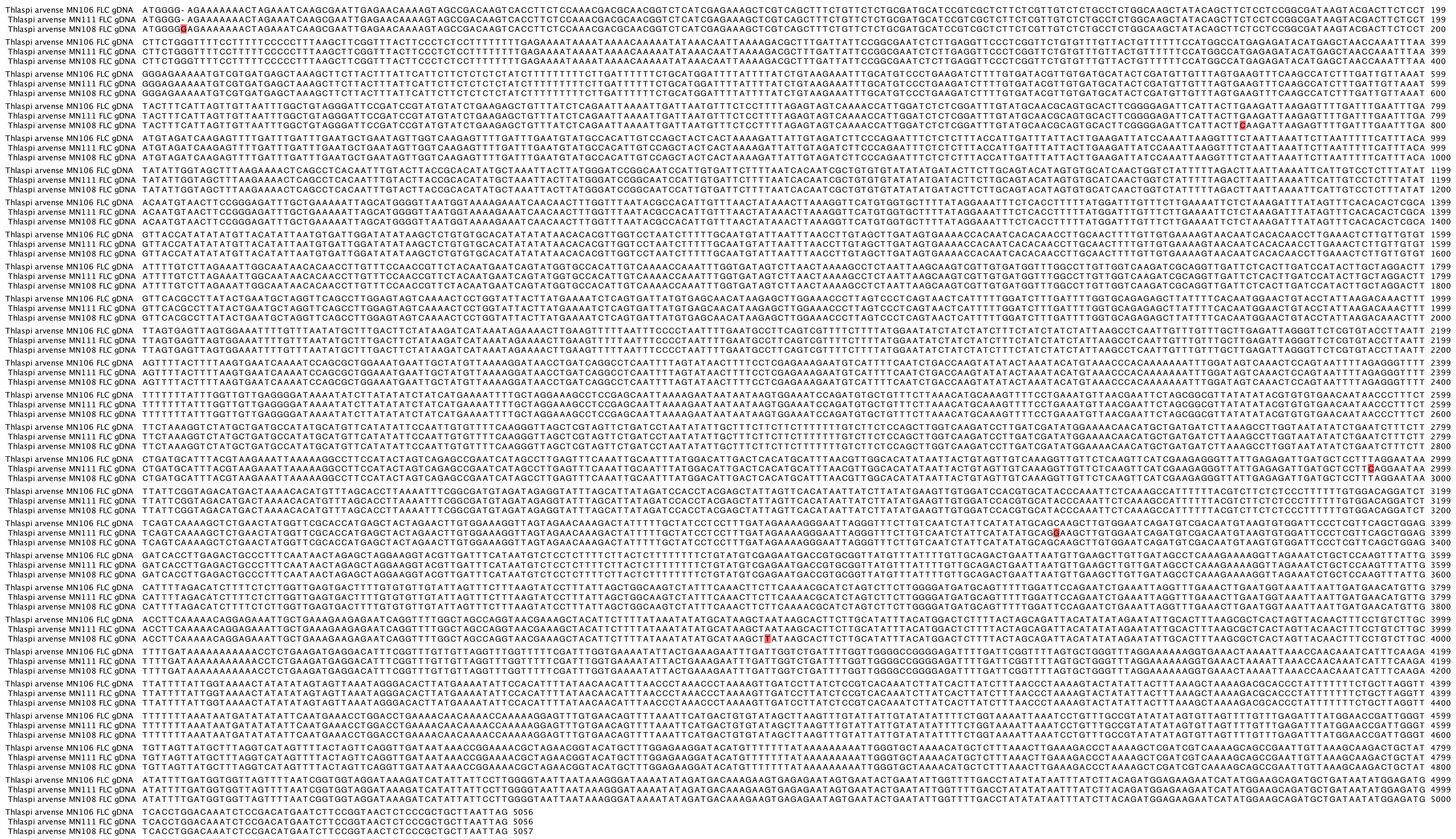
Thlaspi arvense MN106 FLC gDNA
Thlaspi arvense MN111 FLC gDNA
Thlaspi arvense MN108 FLC gDNA
