# Supplementary material for: Spring flowering habit in field pennycress (Thlaspi arvense) has arisen multiple independent times
Source: Plant Direct. 2018 Nov 15;2(11):e00097. doi: 10.1002/pld3.97 (PMC6508777; doi:10.1002/pld3.97)
Supplement: Supplementary file 7 [file PLD3-2-e00097-s007.pdf]

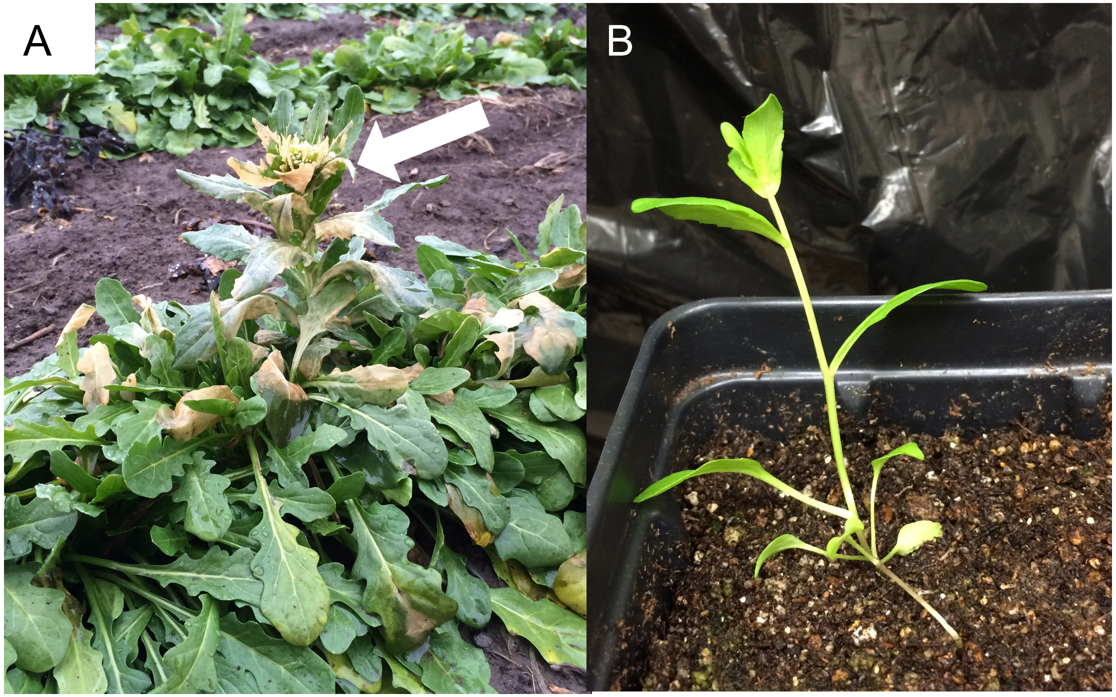

C

MN106 ATGGGGAGAAAAAAACTAGAAATCAAGCGAATTGAGAACAAAAGTAGCCGACAAGTCACCTTCTCCAAACGACGCAACGGTCTCATCGAGAAAGCTCGTCAGCTT

M G R K K L E I K R I E N K S S R Q V T F S K R R N G L I E K A R Q L

flc-**α** ATGGGGAGAAAAAAACTAGAAATCAAGCGAATTGAGAACAAAAGTAGCCGATAA

M G R K K L E I K R I E N K S S R *
