# Supplementary material for: Spring flowering habit in field pennycress (Thlaspi arvense) has arisen multiple independent times
Source: Plant Direct. 2018 Nov 15;2(11):e00097. doi: 10.1002/pld3.97 (PMC6508777; doi:10.1002/pld3.97)
Supplement: Supplementary file 8 [file PLD3-2-e00097-s008.pdf]

Accession	Read Length	Total bp (pre QC)	Total bp (post QC)	Number of Paired-End Reads (pre QC)	Number of Paired-End Reads (post QC)
MN111	2x100 bp	33,778,738,556	27,043,767,076	167,221,478	158,393,618
MN108	2x100 bp	33,807,299,538	27,179,512,142	167,362,869	159,141,481
Ames22461	2x125 bp	17,321,171,868	15,290,002,168	68,734,809	66,483,245
PI633414	2x125 bp	19,449,317,664	17,133,004,749	77,179,832	74,538,343
PI633415	2x125 bp	18,915,382,080	16,469,793,673	75,061,040	71,747,344
PI650284	2x125 bp	17,580,974,544	15,158,595,197	69,765,772	66,056,660
PI650287	2x125 bp	16,589,921,544	14,661,876,555	65,833,022	63,751,658
flc-α (c.52C>T in MN106 background)	2x125 bp	17,220,139,020	15,027,555,931	68,333,885	65,449,912
