# Supplementary material for: Spring flowering habit in field pennycress (Thlaspi arvense) has arisen multiple independent times
Source: Plant Direct. 2018 Nov 15;2(11):e00097. doi: 10.1002/pld3.97 (PMC6508777; doi:10.1002/pld3.97)
Supplement: Supplementary file 9 [file PLD3-2-e00097-s009.pdf]

Name of oligo	Sequence (5' - 3')
TaFLC_1_Forw	CCGAGGAAGAAAAAGTAGATAGAGACA
TaFLC_1_Rev	GAAGCTTAAAGGGGGAAAAAGGAA
TaFLC_2_Rev	CGAACCATAGTTCAGAGCTT
TaFLC_2_Forw	ATAGTGTGCATCAACTGGTC
TaFLC_3_Rev	GCTAATTTTTCAGCAAATCTCCCG
TaFLC_4_Forw	GCGACGGTGAATATGGAGTTGG
