# Supplementary material for: Spring flowering habit in field pennycress (Thlaspi arvense) has arisen multiple independent times
Source: Plant Direct. 2018 Nov 15;2(11):e00097. doi: 10.1002/pld3.97 (PMC6508777; doi:10.1002/pld3.97)
Supplement: Supplementary file 10 [file PLD3-2-e00097-s010.pdf]

Name	Allele	Growth Habit	Source Location
MN106ref	WT	WA	Minnesota
MN111	WT	WA	New York
MN108SA	*flc-A*	SA	Minnesota
Ames31489	*flc-A*	SA	Saskatoon, Canada
MN131	*flc-A*	SA	Howard Springs, MT
Ames31491	*flc-A*	SA	Canada
Ames29512	*flc-B*	SA	Ohio (USDA line - plotted in middle of Ohio)
Ames31024	*flc-B*	SA	Colorado
Ames31490	*flc-B*	SA	Saskatoon, Canada
Ames31492	*flc-B*	SA	Saskatoon, Canada
Ames31500	*flc-B*	SA	Alberta, Canada
MN121	*flc-B*	SA	Roseau, MN
MN123	*flc-B*	SA	Roseau, MN
MN124	*flc-B*	SA	Roseau, MN
MN125	*flc-B*	SA	Roseau, MN
MN129	*flc-C*	SA	Howard Springs, MT
MN133	*flc-C*	SA	Howard Springs, MT
MN134	*flc-C*	SA	Howard Springs, MT
MN135	*flc-C*	SA	Howard Springs, MT
Ames22461	*flc-C*	SA	Slawinek, Lublin, Poland
PI633415	*flc-C*	SA	Belgern, Germany
PI650284	*flc-C*	SA	Kefferhausen, Germany
PI650285	*flc-C*	SA	Leipzig-Mockau, Saxony, Germany
PI650286	*flc-C*	SA	Groitzsch, Saxony, Germany
PI650287	*flc-C*	SA	Molsheim,Bas-Rhin, France
PI633414	*flc-D*	SA	Wachstedt, Germany
