# Supplementary material for: Spring flowering habit in field pennycress (Thlaspi arvense) has arisen multiple independent times
Source: Plant Direct. 2018 Nov 15;2(11):e00097. doi: 10.1002/pld3.97 (PMC6508777; doi:10.1002/pld3.97)
Supplement: Supplementary file 11 [file PLD3-2-e00097-s011.docx]

Dear Dr. Seguí-Simarro and Dr. Fortes:

We are happy to submit a revised version of our manuscript ‘Spring flowering habit in field pennycress (*Thlaspi arvense*) has arisen multiple independent times’. We sincerely appreciate your attention to this manuscript, as well as the comments and suggestions provided by the two anonymous reviewers. We have made several changes to address the points brought up by the reviewers in this revised version and include both a tracked-changes and clean version of the manuscript attached, as well as point-by-point response below.

We believe we’ve addressed the concerns brought up by both reviewers – which have certainly improved the clarity of the manuscript, and hope this work will now be acceptable for publication in Plant Direct. We look forward to your response.

Sincerely,

M. David Marks

Professor

Department of Plant and Microbial Biology

University of Minnesota

Reviewer #1: 
This is a clearly presented study that will be of interest to those who study the genetic basis of flowering time variation. The work is straightforward and I have no criticisms of the experimental results or their presentation. 

In the Introduction, the authors describe the potential utility of pennycress as a fall cover crop that will yield an oil crop in the spring, and that key to this will be flowering and seed harvest before a summer crop of soybean needs to be planted. The authors then discuss their findings on the genetic basis of a pennycress SUMMER-annual habit (loss of FLC) but then in the Discussion note this work may contribute "towards the goal of developing an elite WINTER annual pennycress variety that flowers rapidly in the spring to fit within the short growing season of northern climates." Some readers may find this minor summer- v winter-annual issue confusing. Perhaps the authors might want to come full circle and note why one would not want to develop a summer annual as a cover crop and whether or not "tweaking" FLC might lead to one component of a more "elite" winter annual cultivar. However, the paper is nice as is and I would not make this suggestion a requirement. 

We appreciate Reviewer #1’s comments and suggestions. We have added clarifying statements in the last discussion section (Towards an understanding of flowering time control in pennycress) to close this logical loop regarding how understanding FLC’s role in the summer/winter annual habit will help in breeding winter annual lines.

Reviewer #2: 
The authors describe the linkage of an early flowering phenotype in European pennycress to several mutations in the FLC gene. The experiments are of a good quality and the evidence presented supports the role of the FLC gene in the early flowering phenotype. However, some of the conclusions and the points discussed should be qualified because they are somewhat speculative. 

The authors present the following evidence for the involvement of FLC in the early flowering phenotype: 
0) FLC is a candidate gene found to be involved in other species 
1) A mutation in FLC in a early spring mutant 
2) The segretation of the said mutation in an F2 population 
3) An independent mutation in FLC in an early flowering EMS line 
4) Several alleles from wild early spring mutants 

Evidence 2 is definitive in setteling the linkage of the phenotype to the FLC locus in that family and together with evidences 0 and 3 is highly suggestive of the causal role of the mutations in FLC in the early flowering phenotype. Despite this wealth of evidence we cannot be completely sure of the involvement of the FLC gene, it could be argued, although this would be highly unlikely, that some other gene close to FLC could be responsible for the phenotype. This could only be settled by recreating the mutation in a winter accession like MN111. I am not suggesting that the authors should carry out such an experiment, just that it should be clear in the text that, although the evidences presented are highly suggestive of the role of FLC they do not completely settle the question. 

We thank Reviewer #2 for their comments and suggestions to improve the clarity and conclusions of this manuscript. We certainly agree that to complete the functional characterization of FLC’s role in pennycress vernalization and flowering, additional experiments including targeted mutations of FLC in a winter annual background like MN111 or transgenic experiments to express functional copies of the winter annual FLC mRNA in spring annual backgrounds of either pennycress or Arabidopsis would be needed. However, we believe that the multiple layers of evidence we present in this manuscript – particularly the identification of an EMS-mutation derived allele, along with the conserved function of FLC and it’s orthologs in closely related Brassicaceae species make the scenario of a tightly linked gene near FLC incredibly unlikely, although not impossible. As such, we have added these points of clarification to the section ‘Towards an understanding of flowering time control in pennycress’ at the end of the discussion section.

Unfortunately, there are more problems with evidence 4 and with the conclusions derived from it. The authors try to look for association between mutations in the FLC gene with the early flowering phenotype by looking for mutated alleles in early flowering accessions. This is methodologically dubious. The proper way to do it is to look for the alleles both in early flowering accession and in winter accessions. If we only sequence the FLC gene in the mutants we cannot conclude that the mutations found are associated with the phenotype. Other hypotheses could be suggested. For instance, the gene sequence could be in fact a pseudogene likely to accumulate mutations. If this pseudogene would be located close to the real gene responsible for the phenotype we would get exactly the results that we have observed. I am not implying that this pseudogene hypothesis is likely, I am just saying that the proper way to establish an association between a phenotype and a gene is to check both wild and mutant accessions. 

This is certainly a valid point brought up by Reviewer #2. We apologize if our explanation of the logic and order of things was unclear in the first version of the manuscript. Ultimately, we used WGS to identify these candidate mutations in the spring annuals, and then designed the PCR/amplicon sequencing assays to screen the wider germplasm pool. Included in this wider screening, which was not presented in this manuscript for clarity and succinctness, were all USDA PI accessions plus the UMN germplasm collections. Thus, we did indeed genotype all winter and spring annual accessions in the collection. However, the field-based phenotyping data for the spring/winter annual habit for a several of these lines suggested either segregating spring/winter phenotypes, or contaminated seed lots. Thus, we did not present this dataset in this manuscript. Using this initial field-based screen, we focused on the lines presented here, whose phenotypes were separately confirmed in growth chamber conditions.

I am confused regarding the alleles found in the wild spring mutants. The authors say: "only the flc-B and flc-D alleles were identified in the seven spring annual European accessions tested here" but I think that the figures and tables show that the alleles found in Europe are flc-C and flc-D. I guess that this is a minor mistake.

This was indeed an oversight that was carried over from an old version of the manuscript. It has been corrected (ca. line 426)

The authors propose an hypothesis regarding the population history and the migration of this species to America based on the allele distribution found in the wild species. The proposed scenario is likely, but there could be other proposals. As the authors recognize, much more data would be required to be certain about the population history. So this section of the discussion is somewhat speculative. I don't have any problem with discussing the proposed hypothesis, but it should be emphasized that this is just one possibility, maybe even the most parsimonious explanation given the data, but just one. So I would reduce the relevance given to this issue in the discussion.

We acknowledge the limitations of the current dataset in fully answering this question. We have added several qualifying statements to the discussion section “Global distribution of FLC alleles and the introduction to North America”. As we discuss ca. line 454, additional sequencing experiments of these populations can help clarify the relationship of these European and North American lines. We have already performed genotyping-by-sequencing of our collection, which is being used to address these relationships and will be presented in a follow-up manuscript.
